# Bilberry and Sea Buckthorn Leaves and Their Subcritical Water Extracts Prevent Lipid Oxidation in Meat Products

**DOI:** 10.3390/foods9030265

**Published:** 2020-03-02

**Authors:** Sari Mäkinen, Jarkko Hellström, Maarit Mäki, Risto Korpinen, Pirjo H. Mattila

**Affiliations:** 1 Natural Resources Institute Finland, Production Systems, Humppilantie 7, 31600 Jokioinen, Finland; sari.makinen@luke.fi (S.M.); jarkko.hellstrom@luke.fi (J.H.); maarit.maki@luke.fi (M.M.); 2 Natural Resources Institute Finland, Production Systems, Tietotie 2, 02150 Espoo, Finland; risto.korpinen@luke.fi; 3 Natural Resources Institute Finland, Production Systems, Itäinen Pitkäkatu 4, 20250 Turku, Finland

**Keywords:** natural antioxidants, plant ingredients, subcritical water extraction, lipid oxidation, meat

## Abstract

The aim of this study was to find new sustainable, Nordic natural antioxidant sources, develop subcritical water extraction (SWE) process for recovering the antioxidant compounds from the most potential raw materials, and to test their antioxidative effects in meat products. The antioxidant capacities of water and 50% ethanol (aq) extracts of 13 berry, grain, and horticultural plant materials as well as hexane/ethanol extracted stilbene fractions from pine heartwood and spruce inner bark were measured in hydrophilic and lipophilic systems. Tree, bilberry leaf (BL), and sea buckthorn leaf (SBL) extracts showed the highest antioxidant capacities. BL and SBL were selected for the development of SWE. The optimal conditions for recovering maximal antioxidative capacities were 110 °C/1 min for SBL and 120 °C/1 min for BL. Dried BL and SBL and the respective optimized subcritical water extracts were applied in chicken slices and pork sausage, and their ability to prevent lipid oxidation was evaluated during 8 and 20 days storage, respectively, at 6 °C. All tested plant ingredients effectively prevented lipid oxidation in the products compared to the control samples. Sensory acceptance of the plant ingredients was good, especially in the chicken product. To our knowledge, this is the first study to assess the antioxidant effects of SW extracted berry leaves in meat products.

## 1. Introduction

Meat is a staple food, providing proteins of high nutritional value and a high content of essential minerals and B vitamins [1]. However, meat lacks antioxidants and it is, therefore, susceptible to oxidative changes. Processing, such as grinding, exposes the muscle surface to the air and the lipid membranes to metal oxidation catalysts [2]. Oxidation processes cause deterioration in the flavor, texture and color of meat, induce the development of toxic compounds and loss of nutrients, and reduce shelf life [3]. Antioxidants are used to delay, retard, or prevent oxidative reactions in meat products [4]. The antioxidants added in meat products are mainly synthetic, but due to the current trend to avoid or minimize the use of synthetic food additives, studies to identify novel and natural extracts with potential applications for meat and meat products are needed [5,6,7]. The use of antioxidative plant extracts can be of great benefit also for human health.

Various plant sources have been studied as antioxidants in meat and other products [4,6,8,9]. However, the information of the potential of Nordic plants such as Nordic berry fruit and leaves, trees, grains, and wild edible plants is scarce. It is known that especially Nordic berry fruit and leaves are excellent sources of phenolic compounds such as phenolic acids, flavonoids, and tannins which can act as both primary and secondary antioxidants [9,10,11]. Coniferous trees are also abundant, but neglected sources of structurally similar polyphenols as in berries. Spruce inner bark contains mainly stilbene glucosides (astringin, isorhapontin and piceid) [12], while pine heartwood contains mainly stilbene aglycones (pinosylvin and pinosylvin monomethyl ether) [13].

Efficient extraction of the antioxidants from their natural sources, along with establishing their *in vivo* and *in producto* antioxidant activity, has been a great challenge for researchers [9]. Subcritical water extraction (SWE) is a new, promising extraction method for bioactive compounds. Subcritical water is defined as the water that maintains its liquid state under adequate pressure at temperature between the boiling point 100 °C and critical point 374 °C. Supercritical water has special properties to extract both polar and non-polar analytes. SWE is a green, safe technology which can result in high quality products with lower production cost and higher efficiency [9,14]. 

The aim of this study was to find new sustainable and effective natural, Nordic antioxidant sources, develop SW extraction methods for the most potential raw materials to extract their antioxidative fractions, and test the effects of the materials and their SW extracts in meat products. To our knowledge, this is the first study to assess the antioxidant effects of SW extracted berry leaves in meat products.

## 2. Materials and Methods 

### 2.1. Plant Materials 

13 different samples were collected during 2015–2016, including blackcurrant (*Ribes nigrum*), chokeberries (*Aronia melanocarpa/mitchurinii*), rosehips (*Rosa rugosa*), blackcurrant juice press cake, the hulls of buckwheat (*Fagopyrum escolentum*), Scots pine heartwood (*Pinus sylvestris*), Norway spruce inner bark (*Picea abies*) and the leaves of sea buckthorn (SBL, *Hippophae rhamnoides*), lingonberry (*Vaccinium vitis-idaea*), bilberry (BL, *Vaccinium myrtillus*), goutweed (*Aegopodium podagraria*), nettle (*Urtica dioica*) and dandelion (*Taraxacum officinale*).

Some wild samples (goutweed and dandelion) were picked in southern Finland. The other samples were donated by various Finnish companies and producers. Nettle leaves, hulls of buckwheat, BL, pine heartwood and spruce inner bark were air-dried, and the other samples were freeze-dried in the laboratory before analyses. 

### 2.2. Chemicals 

Ethanol (96%) was purchased from Altia (Rajamäki, Finland). Chemicals and reagents used in measuring antioxidant capacity were purchased from Sigma Chemical (Sigma Chemical Co., St. Louis, MO, USA). The chemicals used in the characterization of the spruce inner bark and pine heartwood extracts were pyridine, N,O-Bis(trimethylsilyl)trifluoroacetamide and chlorotrimethylsilane, purchased from Sigma–Aldrich (St. Louis, MO, USA). The standards of phenolic compounds and the chemicals used in the assays were obtained from various manufacturers. Catechin, epicatechin, gallocatechin, epigallocatechin, caffeic acid, chlorogenic acid, ferulic acid, gallic acid, ellagic acid, protocatechuic acid, p-hydroxybenzoic acid, vanillic acid, syringic acid, p-coumaric acid, and sinapic acid were obtained from Sigma Chemical. Procyanidin B2 was from Extrasynthese (Lyon, France). Acetonitrile, methanol, concentrated hydrochloric acid (37–38%), and phosphoric acid (85%) were from J. T. Baker (Mallinckrodt Baker Inc., Utrecht, The Netherlands). Cysteamine and formic acid were from Sigma Chemical (Sigma Chemical Co., St. Louis, MO, USA).

### 2.3. Extraction of Antioxidants with Water and Ethanol-Water at Ambient Temperature 

All samples except pine heartwood and spruce inner bark were extracted using water and 50% ethanol (aq) with a solid/liquid ratio 1:10. Extraction mixtures were homogenized with Ultra-Turrax T25 (IKA GmbH, Breisgau, Ger), followed by ultrasound assisted extraction (VWR USC 2100D, VWR International, Helsinki, Fin) for 30 min (45 kHz). Extracts were centrifuged, filtered and stored at −20 °C prior to antioxidant analysis.

### 2.4. Two-Step Extraction of Tree Materials and Determination of Phenolic Compounds 

The pine heartwood and spruce inner bark extracts were obtained by two-step extraction using hexane and 95% ethanol (aq) according to the previously optimized protocol [15]. A stainless steel extraction cell (Dionex Corp., Sunnyvale (CA), USA) was loaded with raw material powder and extracted with n-hexane at 90 °C, and the residue was again extracted with ethanol/H_2_O (95:5, *v*/*v*) at 100 °C using accelerated solvent extraction equipment Dionex ASE-350 (Dionex Corp., Sunnyvale (CA), USA). The extractions were performed as 3 × 5 min static cycles. The ethanolic extracts were further used and concentrated using a rotary evaporator.

The dry solids content of the extracts was determined gravimetrically, and polyphenols were determined by a GC-MS analysis [16]. Briefly, aliquots of the extracts were evaporated to dryness under an N_2_ stream and silylated by adding 150 μL of a mixture of pyridine, N,O-bis(trimethylsilyl) trifluoroacetamide (BSTFA) and trimethylsilyl chloride (TMCS), at a 1:4:1 (*v/v/v*) ratio, and the mixture was heated in an oven at 70 °C for 45 min. Betulinol (0.02 mg/mL) and heptadecanoic acid (C17:0, 0.02 mg/mL) served as internal standards. The silylated samples were quantified by GC-MS as described earlier [16]. 

### 2.5. Subcritical Water Extraction of Berry Leaves and Determination of Phenolic Compounds 

SWE was developed for BL and SBL using accelerated solvent extraction equipment Dionex ASE 350 (Dionex Corp., Sunnyvale (CA), USA). SWE conditions, i.e., extraction temperature and static extraction time, were optimized with regard to the antioxidant activity of the extracts using response surface modelling with MODDE (BioPAT^®^) chemometrics software. The solid/liquid ratio was set at 1:10. The extracts were frozen at −20 °C immediately after extraction and later lyophilized. 

Raw materials and the optimized SW extracts were analyzed for the content of major phenolics (i.e., phenolic acids and condensed tannins in BL extract, and ellagitannins and condensed tannins in SBL extract) using previously published high performance liquid chromatographic (HPLC) methods [17,18,19]. 

### 2.6. Antioxidant Activity of Plant Extracts In Vitro

The antioxidant activity of the plant extracts was assessed in aqueous phase as radical scavenging capacity using the ABTS [(2,20-azinobis-(3-ethylbenzothiazoline-6-sulfonic acid)] decolorization assay [20] with slight modifications [21]. The results are expressed as Trolox equivalent antioxidant capacity (TEAC) values, describing the capacity of the samples to scavenge radicals in mg dm/mL in comparison to Trolox.

The susceptibility of the plant extracts to inhibit lipid oxidation was assessed in a lipid phase with a liposome model [22] with some modifications. Briefly, soybean phosphatidylcholine liposomes were prepared according to Ursini et al. [23]. Liposomes were stored at 4 °C at least one week prior to the study to increase the lipid hydroperoxide levels. The lipid oxidation reaction was conducted as described earlier [24,25]. Briefly, liposomes (100 µL) were mixed with sample, buffer (50 mM K-phosphatebuffer pH 7.4, 100 mM glysine, 450 μM ascorbic acid) and oxidative agent (150 µL of 1 mM ADP in 25 μM FeCl_3_) at various sample concentrations. The suspension was allowed to react for 48 h at room temperature in the dark. Consequently, the concentration of the thiobarbituric acid reactive substances (TBARS) formed during the liposome oxidation was determined by a color reaction with thiobarbituric acid (TBA) and butylated hydroxytoluene (BHT). The color reaction was performed by mixing the oxidized liposome suspension with trichloroacetic acid (TCA)/TBA solution (0.375% TBA, 2.25% TCA in 0.25 M HCl) and BHT (2% BHT in Methanol) and consequent incubation in a boiling water bath for 30 min. The solution was cooled to room temperature and centrifuged at 1710× *g* for 10 min. Aliquots, 30 µL, of the supernatants, were injected into an Agilent 1100 HPLC-DAD with a SunFire C18 column (4.6 mm × 150 mm, 5 μm particle size, Waters). Samples were eluted with a linear gradient (6–99% in 30 min) of acetonitrile in 0.05% trifluoroacetic acid, and the effluent was monitored at 532 nm. The concentration of malondialdehyde (MDA) was calculated against the MDA-TBA standard curve (12.5–800 μM). Samples were analyzed in triplicates.

Results from the liposome model are presented as an inhibition efficiency ratio (IER) describing the inhibition percentage produced with a sample concentration of 1 µg dm/mL. For the samples with the highest antioxidant potential (spruce inner bark and pine heartwood extracts) and for BL and SBL SW extracts the IC50 values were measured. The IC50 value indicates the concentration of a sample µg dm/mL needed to inhibit 50% of the lipid oxidation in the liposome model. The IC50 value was calculated using a linear regression from a plot inhibition percentage versus sample concentration µg dm/mL.

### 2.7. Application of Berry Leaves and Their Subcritical Water Extracts in Chicken Marinades and Pork Sausages 

The capacities of dried and homogenized BL and SBL and their SW extracts to prevent lipid oxidation in meat products were tested in sausage and marinated chicken leg slices. The concentrations used were selected according to IC50 values and preliminary tests. In the sausage test, the basic sausage mass contained pork meat 75%, water 25%, white pepper 2g/kg mass, salt 16.6 g/kg mass, and diphosphates (E450) 3g/kg mass, and there were eight treatments (Table 1). Treatment 1 served as a negative control containing only basic mass, treatments 2–7 contained test materials and treatment 8 served as a commercial (positive) control containing NaNO_2_ and ascorbic acid. Three 400 g sausages were prepared for each treatment by casing in commercial synthetic sausage skin, heat-treated to an inner temperature of 72 °C, cooled in running cold water and stored overnight in a refrigerator below 6 °C. On the following day, the sausages were cut into small cubes (1–1.5 cm^3^) and pooled according to the treatments. The amount of 100 g of each treatment were taken for sensory analysis, and the remaining pools were divided into 80 g portions which were stored in plastic bags below 6 °C in a refrigerator until lipid oxidation analysis.

In the marinated chicken leg test, there were also eight treatments (Table 2). In each treatment, 400 g of chicken leg slices were divided into 70 g portions and mixed in a plastic bag with 30 g of the marinades described in Table 2. The basic marinade contained rapeseed oil 52%, sucrose 11%, salt 6%, water 21%, and 6% commercial spirit vinegar 10% (aq). Treatment 1 was a negative control containing only the basic marinade, treatments 2–7 contained test materials and in treatment 8 there was no marinade at all. After two hours’ stabilization at 6 °C, 100 g-bags of each treatment were taken for sensory analysis. The remaining samples were stored at 6 °C in a refrigerator until analysis of lipid oxidation.

### 2.8. Sensory Evaluation

A sensory evaluation of the marinated chicken slices and sausages was conducted readily after preparation by 5 male and 5 female panelists. The samples were labelled with 3-digit random numbers. The sausages were evaluated in two groups of 4 and 5 samples per session. Sausages with 0.2% BL extract were evaluated in both sessions. Marinated chicken slices were fried before sensory evaluation using a Tefal ActiFry low-fat fryer. Sensory evaluation was conducted in two groups of 4 samples per session. 

Panelists were given three slices per treatment and asked to evaluate on a scale with fixed extremes from 0 to 5. The evaluated parameters for preference were color (0 = unpleasant, 5 = tempting), flavor and overall acceptability (0 = poor, 5 = excellent). Each point marked was converted to a numerical value as a distance from 0. The most preferred treatments were estimated by ranking the sensory attribute median values. A nonparametric Kruskal-Wallis H test was used to determine if there were statistically significant differences between treatments. The statistical analysis was performed using (IBM SPSS Statistics for Windows, Version 25.0. Armonk, NY: IBM Corp.).

### 2.9. Oxidation of Lipids in the Meat Products

The capacity of the BL and SBL and the respective SW extracts to prevent lipid oxidation in the products was assessed by the prevention of TBARS formation during storage. The TBARS in sausage samples were measured after 10 and 20 days of storage. The lipid oxidation status of the marinated and sliced chicken legs was measured after 4 and 8 days of storage. The lipid oxidation statuses as TBARS levels of the sausages and marinated chicken leg slices were measured using a specific HPLC method, described previously in Section 2.4. Prior to the analysis, the sausage and marinated chicken slice samples were subjected to alkaline hydrolysis to release MDA from meat proteins. First, samples were homogenized using Ultra-Turrax T25 (IKA GmbH, Breisgau, Germany), and four 100 mg subsamples of each homogenized sample were taken for alkaline hydrolysis. The hydrolysis was conducted by mixing the 100 mg subsamples with 200 µL of 1.5 M NaOH and incubating the suspensions in a 60 °C water bath for 30 min. After the hydrolysis, 1 mL of 0.05 M sulfuric acid and 0.5 mL of 20% (*w/v*) TCA were added, and the precipitated proteins were separated by centrifugation (3000 rpm, 10 min). The supernatants were then reacted with TBA to form MDA-TBA adducts with pink pigment and analyzed with HPLC, as previously described in Section 2.4. Chromatographic analyses were performed in duplicate from each of the subsamples (*n* ≥ 8). Results are expressed as mean ± SD. An independent Student’s *t*-test was used to compare the effects of the plant ingredients on the TBARS formation during storage.

**Figure 1:**
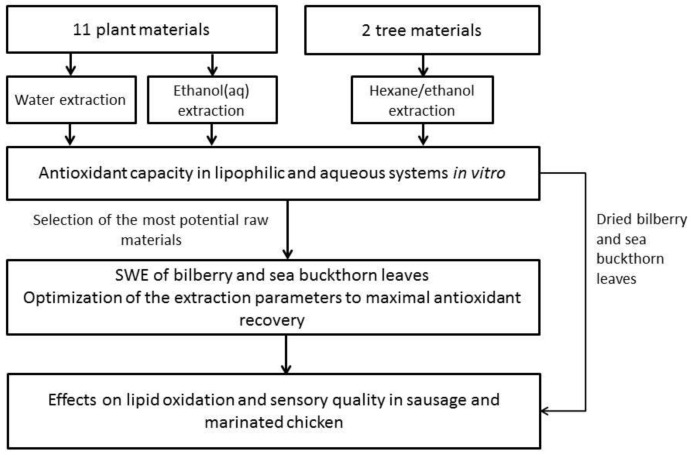
The flow diagram of the study. Chemical-free methods (water and SWE) were preferred for the recovery of antioxidants.

The flow diagram of the study is in Figure 1.

## 3. Results and Discussion

### 3.1. Antioxidant Activities of Water, Ethanolic (aq) and Hexane/Ethanol Extracts In Vitro

**Figure 2:**
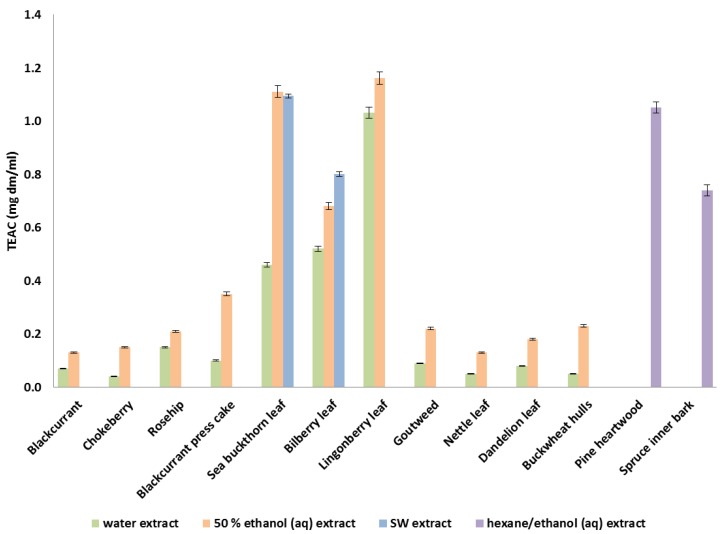
Radical scavenging activity as TEAC (mg dm/mL) of the selected plant extracts.

The plant extracts showed high variability in antioxidant potential by means of radical scavenging capacity as well as inhibition of lipid oxidation. The highest radical scavenging potential as TEAC (mg dm/mL) was observed in the ethanolic extracts of the leaves of sea buckthorn (1.1 ± 0.02), lingonberry (1.2 ± 0.02) and bilberry (0.7 ± 0.1), and in the hexane/ethanol extracted pine heartwood (1.1 ± 0.02) and spruce inner bark (0.75 ± 0.01; Figure 2). Extraction with water at ambient temperature resulted in lower radical scavenging activities compared with 50% ethanol extraction with all tested raw materials. However, with bilberry and lingonberry leaves, the difference was minor and high radical scavenging potential was also observed in the water extracts (Figure 2). The radical scavenging activities of the studied plant materials were at the same level as reported for e.g., grapevine leaves [26], while the commonly known antioxidant herbs rosemary (*Rosmarinus officialis*) and thyme (*Thymus vulgaris*) have shown slightly higher efficacies [27]. 

**Figure 3:**
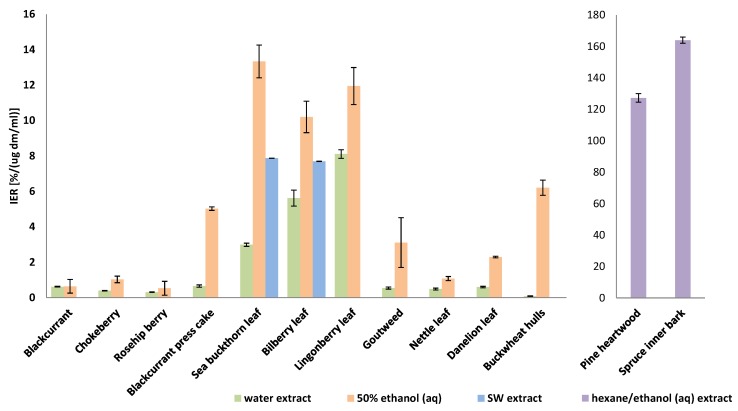
Capacity of the selected plant extracts to prevent lipid oxidation in a liposome model in IER values [%/(ug dm/mL)].

In the liposome model, chokeberry, blackcurrant, and rosehip showed almost no efficacy against lipid oxidation, whereas the leaf extracts of sea buckthorn, bilberry and lingonberry possessed high efficacies (Figure 3). However, among the samples, pine heartwood and spruce inner bark extracts showed superior capacity to prevent lipid oxidation. The IER values [%/(ug dm/mL)] of pine heartwood extract and spruce inner bark extract were 127 ± 4 and 164 ± 5, respectively, while the corresponding values for the berry leaf ethanolic extracts varied from 10 ± 0.3 (BL) to 13 ± 0.4 (SBL). The concentration of sample needed to inhibit peroxidation by half was measured to further characterize the antioxidant efficacy of the samples with the highest potential. The IC50 values of pine heartwood and spruce inner bark extracts were very low 0.7 × 10^−3^ and 0.6 × 10^−3^ µg dm/mL, respectively, indicating that picogram level concentrations of the tree extracts are enough to inhibit 50% of the lipid peroxidation in the liposome model.

In the literature, lipid oxidation inhibition capacity values of 25–51% at a sample concentration of 1.4 µg dm/mL have been reported for the phenolic extract of raspberry, lingonberry and bilberry [28]. The plant extracts in the present study, especially the pine heartwood and spruce inner bark extracts showed higher efficacies. A high antioxidative power of tree extracts was expected, because they were rich in stilbenes (see Section 3.3), which are known to be effective phenolic antioxidants [29,30,31]. Lower IER and higher IC50 values have been reported e.g., for proteinaceous extracts of rapeseed and linseed [22,32]. The results indicate that the plant extracts of the present study possess significantly higher antioxidant capacity compared with the proteinaceous extracts. 

### 3.2. Subcritical Water Extraction for Bilberry and Sea Buckthorn Leaves and Antioxidant Activities of the Extracts

Because BL and SBL ethanolic extracts proved to be highly antioxidative they were selected as raw materials for the development of SW extraction processes. Lingonberry leaves had even higher antioxidative efficacy but the dominant phenolic compound in lingonberry leaves is ß-p-arbutin which can have some adverse effects in higher doses limiting its usage in food applications [10,33]. In addition, tree extracts had high antioxidant capacity, but their extraction processes have been optimized earlier [15]. 

The SWE parameters (temperature and static extraction time) were optimized for antioxidant recovery with response surface modelling. The optimal conditions predicted with MODDE (BioPAT^®^) chemometrics software were 110 °C/1 min for SBL and 120 °C/1 min for BL. Optimal conditions were applied for the samples, and the antioxidant activities measured from the extracts followed the predicted values very well. Using these optimized conditions, the radical scavenging activities of BL and SBL were 0.8 ± 0.008 and 1.1 ± 0.006 TEAC (mg dm/mL), respectively (Figure 2). These values were of the same magnitude or even higher than those of ethanolic extracts and clearly higher in comparison to conventional water extracts (Figure 2). The ability of SW extracts to inhibit lipid peroxidation was somewhat lower than that of ethanolic extracts but clearly higher than that of conventional water extracts (Figure 3). The concentration of sample needed to inhibit peroxidation by half was measured to estimate how much of the extracts are needed for meat product tests. IC50 values were 9.2 × 10^−3^ µg dm/mL for BL SW extract and 4.8 × 10^−3^ µg dm/mL for SBL SW extract. 

Previously, Kumar et al. [34] used SW extraction to recover antioxidant compounds from sea buckthorn leaves. Extraction temperatures of 100, 150 and 200 °C with 15 min extraction time were used, and as a result, phenolic compounds were recovered most efficiently at 150 °C. According to Shitu et al. [14] SWE can be successfully applied in extracting phenolic compounds from fruit peel, shell, seed, and food matrices, among others. Naturally, SWE conditions vary according to the material. Singh and Saldaña [35] extracted phenolic compounds from potato peels at 180 °C with an extraction time of 30 min. Tunchaiyaphum et al. [36] used conditions of 180 °C, 90 min, a solid to water ratio of 1:40 and pH 4 for mango peels. The optimal extraction parameters (the highest ABTS radical scavenging activity) for sea buckthorn seed residue extracts were 120 °C, 36 min, and a water to solid ratio of 20 [37]. In the present study the optimal time for antioxidant recovery was 1 min which is much shorter than those used in the previous literature [34,35,36,37]. In general, high temperatures and prolonged extraction times tend to improve the extractability of compounds, but they can also induce the degradation of heat sensitive molecules such as many natural antioxidants.

### 3.3. Polyphenol Contents in the Leaf and Tree Extracts

Polyphenol contents were analyzed from the tree extracts of the previously optimized 2 step process and the leaf extracts of the SW processes optimized in the present study. The concentrated pine heartwood extract (dry solids content 83.10 mg/mL) contained two stilbene compounds, pinosylvin (PS) 11.23 ± 0.40 mg/mL and pinosylvin monomethyl ether (PSMME) 9.27 ± 0.94 mg/mL. The PS/PSMME ratio was 1.2 which was in accordance with the study by Willför et al. (2003) [13]. According to Willför et al. [13] the contents of PS and PSMME in Scots pine heartwood varies 3.7–5.5 mg/g and 5.1–6.3 mg/g, respectively. The concentrated spruce inner bark extract (dry solids content 49.40 mg/mL) contained three stilbene glucosides, piceid (0.98 ± 0.02 mg/mL), isorhapontin (8.14 ± 0.10 mg/mL) and astringin (6.38 ± 0.45 mg/mL). 

Phenolics contents in berry leaves were 5.58 ± 0.25 g/100 g dw of phenolic acids (mostly caffeoyl-quinic acids) and 3.71 ± 0.28 g/100 g dw of condensed tannins for BL, and 8.13 ± 0.23 g/100 g dw of ellagitannins and 1.52 ± 0.06 g/100 g dw of condensed tannins for SBL. The dried BL SW extract contained phenolic acids 12.9 ± 0.1 g/100 g (recovery of 78%) and condensed tannins 5.6 ± 0.3 g/100 g (recovery of 47%). The dried SW extract of SBL contained 13.3 ± 0.4 g/100 g of ellagitannins (recovery of 56%) and 3.4 ± 0.2 g/100 g of condensed tannins (recovery of 76%). In BL extract the recovery of total phenolics was 65% and in SBL extract it was 59%. According to Tian et al. [10] the total content of phenolics was consistently higher in leaves than in berries in 13 plant species. Sea buckthorn, lingonberry, and bilberry leaves were richest in phenolic compounds in this order. Ellagitannins dominated in sea buckthorn leaves and caffeoylquinic acids in bilberry leaves. These findings accorded well with our results. 

### 3.4. Antioxidative Effects of Berry Leaves and Their SW Extracts in Marinated Chicken and Pork Sausage

**Figure 4:**
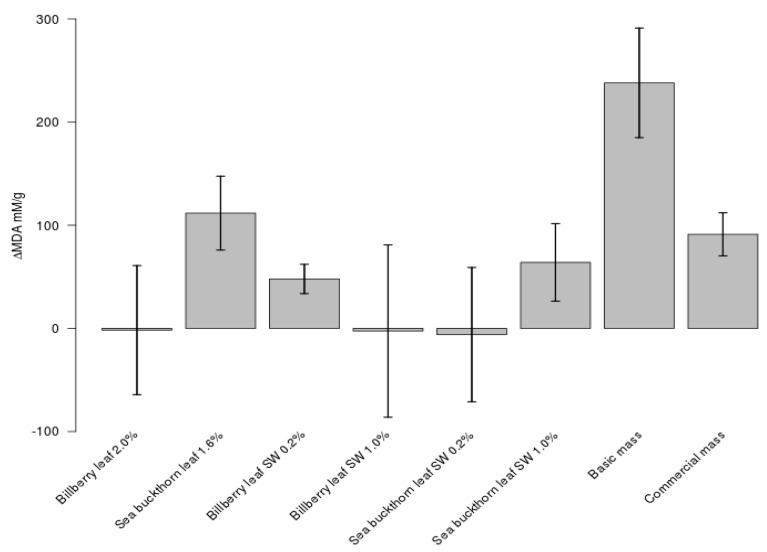
Effects of bilberry and sea buckthorn leaves and their SW extracts on lipid peroxidation in sausage samples during 20 days of storage.

Dried BL and SBL as well as their SW extracts were chosen to the meat product test because they were among the most antioxidative materials and their taste and safety properties were acceptable (see Section 3.1). In sausages, after 20 days of storage, samples amended with BL (2% *w/w*) and BL SW extract (0.2% *w/w*) showed a significantly lower level of TBARS than the commercial sausage mass with nitrite and ascorbic acid (Figure 4). During the 20 days of storage, the TBARS content increased by 94 ± 20 mM/g in the commercial sausage mass, while the respective change in the TBARS for the sausage amended with BL SW extract (0.2% *w/w*) was significantly lower, 54 ± 14 mM/g (*p < 0.08). BL (2% *w/w*) prevented lipid oxidation most efficiently, as no increase in the TBARS level was observed during the 20 days of storage (**p < 0.08). In comparison, the TBARS content of the basic sausage mass prepared without any plant ingredient or nitrite or ascorbic acid increased by 238 ± 24 mM/g during the 20 days of storage. The TBARS level after 10 days of storage were at the same than those after the 20 days of storage except in the basic mass, in which the TBARS content was 172 ± 53 mM/g after 10 days of storage. Altogether, BL and the SW extracts of BL and SBL were the most effective in protecting lipids from oxidation; they could prevent the formation of the TBARS at a significantly higher efficacy than the additives in the commercial sausage mass.

**Figure 5:**
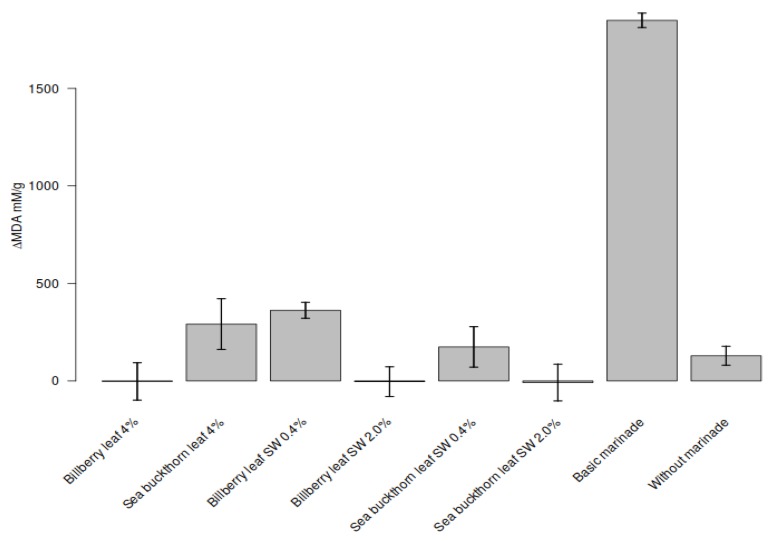
Effects of bilberry and sea buckthorn leaves and their SW extracts on the lipid peroxidation of marinated chicken legs during 8 days of storage.

The capacity of BL and SBL and their SW extracts to prevent lipid oxidation in marinated chicken slices was studied for eight days of storage. The increase in the TBARS content varied from zero (BL and SW extracts of BL and SBL) to 1848 ± 37 mM/g (basic marinade) during the eight days of storage. The TBARS level was significantly lower in the marinated chicken samples amended with plant extracts than in the sample prepared with the basic marinade. All studied plant materials prevented lipid oxidation in marinated chicken, and no statistically significant difference was observed between them during the eight days of storage (Figure 5). The results indicate that BL and SBL, and the respective SW extracts, are potential natural antioxidative agents for preventing lipid oxidation in meat products and therefore provide new potential for developing healthier meat products. 

To our knowledge, neither BL nor SBL or their SW extracts have previously been tested as antioxidants in meat products. However, Nowak et al. [38] studied water extracts of cherry and blackcurrant leaves as preservatives in meat products. These leaf extracts had a good antioxidant effect, and they also enhanced the microbial quality of the pork sausages over 14 days of refrigerated storage. Püssa et al. [39] showed that the ethanol slurry of the juice-free solid residue of sea buckthorn berries inhibited the oxidation of unsaturated fatty acids in cooked chicken and mechanically deboned turkey meat. The polyphenols, mainly flavonols, were responsible for this inhibition. Garrido et al. [40] found that grape pomace extract gained from methanolic extraction + high–low instantaneous pressure was efficient in inhibiting lipid oxidation in pork burgers. Vaithiyanathan et al. [41] evaluated the effect on the shelf life of chicken meat held under refrigerated storage at 4 °C of dipping in pomegranate fruit juice phenolics solution. Pomegranate fruit juice phenolics reduced protein oxidation, inhibited bacterial growth and the products were sensorially acceptable after up to 12 days of refrigerated storage at 4 °C. Huang et al. [42] tested Lotus rhizome knot and leaves extract for raw and cooked porcine and bovine meat. Antioxidant activity was significantly increased in all meat samples with the addition of both extracts, but knots were more effective against lipid oxidation than leaves.

### 3.5. Effects of the Plant Ingredients on the Sensory Properties of the Meat Products

Only the first batches of the meat products were used for sensory analyses to evaluate the sensory properties caused by the ingredients. There was a statistically significant difference in all preference parameters (overall preference *p* = 0.035, color preference *p* < 0.001, flavor preference *p* = 0.010) between treatments of sausages, but not in any preferences of marinated chicken slices (*p* > 0.05). 

Sausages with SBL 1.6% and BL extract 0.2% (A) were nearly as preferred overall as commercial sausage (Table 3). However, sausage with 0.2% BL extract was evaluated twice, and the overall preference scores decreased considerably during the second sensory evaluation session (B). One reason may be that the extract was unevenly distributed in the sausages. It seemed that sausages with 2% of BL and 1% of BL extract, as well as 0.2% and 1% of SBL extracts were found least appealing. Interestingly, the color of almost all sausages was evaluated as more appealing than the color of the sausages prepared from the basic mass without nitrite.

In marinated and fried chicken slices, the differences were less pronounced than in sausages. The overall preference for chicken slices marinated with 4% BL, 4% SBL and 0.4% BL extract were evaluated as similar to the chicken slices using the basic marinade (Table 4). The colors of chicken slices with 4% SBL and 2% BL extract were the best. The flavors were best in slices marinated in 4% SBL and 0.4% BL extract.

A crucial challenge in applying plant extracts in meat products is the color and bitter flavor due to e.g., polyphenols. However, in this study, many of the natural antioxidants tested showed acceptable sensorial properties, and with further product development it may be possible to produce commercial products using them. It seemed that tested leaves and leaf extracts were more suitable for marinade than sausage ingredients. In marinade, they efficiently counteracted oxidation, and their content in the fried product was quite small.

Various natural antioxidants have been shown to exert a positive or negative effect on the color and sensory properties of the meat products [4,9]. For example, cherry and blackcurrant leaf extracts had no negative effects on the sensory attributes of the pork sausages compared with the control sausages [38]. However, the results of Latoch and Stasiak [43] indicated that cooked pork sausages supplemented with mint leaf extract had slightly worse sensory characteristics than the control sausages, although they were still acceptable.

## 4. Conclusions

This study showed that berry leaves, pine heartwood, and spruce inner bark extracts possess superior radical scavenging potential and capacity to inhibit lipid oxidation. SWE emerged as a promising green, chemical-free method for recovering antioxidative compounds from plant materials. BL and SBL, as well as their SW extracts, efficiently prevented lipid oxidation in pork sausage and marinated sliced chicken legs. The results indicate that BL and SBL, and their SW extracts, are potential natural antioxidative agents for preventing lipid oxidation in meat products and therefore provide new possibilities for developing healthier meat products.

## Figures and Tables

**Table 1:** Pork sausage treatments.

Treatment	Content
1	Basic mass
2	Basic mass + 2% bilberry leaf
3	Basic mass + 1.6% sea buckthorn leaf
4	Basic mass + 0.2% bilberry leaf extract
5	Basic mass + 1.0% bilberry leaf extract
6	Basic mass + 0.2% sea buckthorn leaf extract
7	Basic mass + 1.0% sea buckthorn leaf extract
8	Basic mass + NaNO_2_ (0.1 g/kg mass) andascorbic acid (0.6 g/kg mass)

**Table 2:** Chicken leg marinade treatments.

Treatment	Content
1	Basic marinade
2	Basic marinade + 4% bilberry leaf
3	Basic marinade + 4% sea buckthorn leaf
4	Basic marinade + 0.4% bilberry leaf extract
5	Basic marinade + 2% bilberry leaf extract
6	Basic marinade + 0.4% sea buckthorn leaf extract
7	Basic marinade + 2% sea buckthorn leaf extract
8	No marinade

**Table 3:** Medians of preference scores (rank) of sausages (*n* = 10).

Sample	Overall Preference	Color	Flavor
Bilberry leaf extract 0.2% A	3.5 (1)	3.4 (1)	3.5 (1)
Bilberry leaf extract 0.2% B	2.1 (5)	2.5 (4)	2.4 (5)
Bilberry leaf extract 1%	1.8 (8)	2.3 (5)	1.8 (8)
Bilberry leaf 2%	1.7 (9)	1.5 (7)	1.6 (9)
Sea buckthorn leaf extract 0.2%	1.9 (7)	2.0 (6)	2.4 (5)
Sea buckthorn leaf extract 1%	2.1 (5)	0.8 (9)	2.2 (7)
Sea buckthorn leaf 1.6%	3.0 (3)	2.7 (3)	2.7 (3)
Basic mass	2.3 (4)	1.5 (7)	2.5 (4)
Commercial mass	3.2 (2)	3.4 (1)	3.5 (1)

**Table 4:** Medians of preference scores (rank) of marinated broiler slices (*n* = 10).

Sample	Overall Preference	Color	Flavor
Bilberry leaf 4%	3.0 ^1^ (1)	3.3 (3)	2.8 (3)
Seabuckthorn leaf 4%	3.0 ^1^ (1)	3.5 (1)	3.3 (1)
Bilberry leaf extract 0.4%	3.0 ^1^ (1)	2.5 (5)	3.1 (2)
Bilberry leaf extract 2%	2.3 (7)	3.5 (1)	2.5 (4)
Sea buckthorn leaf extract 0.4%	2.5 (6)	2.5 (5)	1.9 (6)
Sea buckthorn leaf extract 2%	2.6 (5)	2.7 (4)	1.8 (7)
Basic marinade	3.0 ^1^ (1)	2.3 (7)	2.0 (5)

^1^*n* = 9.
